# Prospective comparison of econometric, machine learning, and foundation models for forecasting emergency department boarding patients

**DOI:** 10.1038/s44401-025-00054-z

**Published:** 2025-12-16

**Authors:** Lily Poursoltan, Jie Cao, Brian Clay, Beth Trimble, Leah Adrid, Jeffrey Pan, Andrew Chua, John Bell, Christopher A. Longhurst, Kevin Zhu, Karandeep Singh

**Affiliations:** 1 Rady School of Management, University of California, San Diego, CA USA; 2 Joan and Irwin Jacobs Center for Health Innovation, UC San Diego Health, San Diego, CA USA; 3 Mission Control, UC San Diego Health, San Diego, CA USA; 4 Division of Biomedical Informatics, Department of Medicine, University of California, San Diego, CA USA; 5 Division of Hospital Medicine, Department of Medicine, University of California, San Diego, CA USA

**Keywords:** Health care, Mathematics and computing

## Abstract

Emergency department (ED) boarding drives overcrowding, worsens outcomes, and strains hospital operations. Accurate short-term forecasts of boarding volumes can enable proactive resource management. In this prospective study, we compared six forecasting approaches across econometric, machine-learning, and foundation-model paradigms to predict ED boarding volumes up to four days ahead (T + 1–T + 4). Using data from two UC San Diego Health EDs (October 2022–October 2024), models incorporated prior boarding volumes, scheduled surgeries, hospital census, and expert-selected covariates. We evaluated vector autoregression (VAR), extreme gradient boosting (XGBoost), and Google TimesFM, including hybrids (VAR+XGBoost; TimesFM+XReg), against a two-week moving-average baseline. During a four-month validation period, the VAR+XGBoost hybrid achieved the lowest root mean square error—reducing forecast error by 16–42% at La Jolla and 5–19% at Hillcrest—while showing only slight gains over VAR (0–3%). VAR alone performed robustly and outperformed baseline at both sites. The VAR model has been deployed within the health system’s Mission Control to guide proactive interventions such as discharge acceleration and surgical rescheduling. These findings underscore the enduring value of econometric models and demonstrate how forecast-driven decision support can enhance emergency care coordination and system responsiveness.

## Introduction

Emergency department (ED) overcrowding has become a pervasive challenge in health systems worldwide, leading to prolonged wait times, resource strain, and compromised patient care^1,2^. A particularly acute aspect of this overcrowding is ED boarding, a situation in which patients requiring inpatient admission remain physically in the ED due to a lack of available inpatient beds, often waiting in hallways or temporary holding areas^3,4^. This crisis has reached such severity that the American College of Emergency Physicians recently declared ED boarding a national public health emergency^5^. Although The Joint Commission advises that boarding should not exceed four hours due to safety concerns, boarding times have been steadily increasing over recent years, with some cases exceeding 24 hours^3,6^. Prolonged ED boarding contributes to significant downstream risks, including increased mortality, higher rates of intensive care admissions, extended lengths of stay, and more frequent medical errors, all of which compromise patient safety and satisfaction^7–12^. These impacts are especially pronounced when hospital occupancy exceeds 85% to 90%, further straining resources and reducing care quality, while research also links extended boarding times with lower patient satisfaction^4,13^.

Accurate forecasting of inpatient admissions within the ED is essential for mitigating the challenges of ED boarding and overcrowding, especially as hospitals face increased pressure to optimize efficiency and control costs with fluctuating patient volumes^14,15^. Traditional methods for predicting patient volumes, while providing some insights, often lack the precision needed to address the complex and dynamic demands of ED boarding, leading to cascading impacts on ED throughput, inpatient admissions, and surgical scheduling^16^. Better predictions would enable hospital administrators to make proactive resource allocation decisions^17,18^. Models tailored to ED boarding forecasting can inform timely interventions, such as discharging stable patients, facilitating intrasystem hospital transfers, and rescheduling non-urgent surgeries, thus alleviating ED boarding congestion and freeing up critical bed space^14,18^. By better predicting patient inflow, bed demand, and potential bottlenecks, these tools can also enhance resource allocation and staffing decisions. Evidence shows that predictive models improve patient throughput, quality of care, and operational efficiency, underscoring their role as pivotal tools for modern ED operations and patient outcome improvement^14,19,20^.

Commonly used forecasting methods often rely on a single variable without covariates or are designed to capture weekly and linear trends (through seasonality and differencing, respectively) but not nonlinear trends. In a dynamic ED environment where any given factor can lead to a breaking point at which boarding patients accumulate at a greater rate, capturing nonlinearity may be important. The model presented in this study aims to forecast ED boarding volume up to four days in advance by combining a vector autoregression (VAR), an econometrics forecasting approach, with gradient boosting. This framework addresses several critical operational challenges: enabling proactive rather than reactive resource allocation, providing a data-driven foundation for staffing decisions, recommending patient transfer decisions within hospitals, and contributing to more efficient bed management. By prospectively embedding forecasting into daily Mission Control operations, this study illustrates a learning health system approach in which data, predictive models, and operational feedback are continuously integrated. In addition, by examining performance across two hospitals serving diverse populations, the study informs strategies to strengthen system responsiveness and equity in emergency care delivery.

## Results

### Study cohort

The median number of boarding patients was 45.5 (IQR 35.0–63.0) at La Jolla and 12.0 (IQR 7.0–18.0) at Hillcrest. The boarding patients at the La Jolla ED were 60.8 years old (IQR 47.0–74.0), 49.3% female, 4.5% Black, and 23.9% Hispanic, while the patients at Hillcrest ED were 57.5 years old (IQR 44.0-–71.0), 41% female, 10.6% Black, and 29.4% Hispanic (Table 1).

**Table 1:** Demographic Characteristics of Boarding ED Patients

Characteristic	All (*N* = 7111) Mean (SD) or *N* (%)	Boarding La Jolla ED Patients (*N* = 3900) Mean (SD) or *N* (%)	Boarding Hillcrest ED Patients (*N* = 3211) Mean (SD) or *N* (%)
Age	59.3 (18.9)	60.8 (18.2)	57.5 (19.6)
Sex			
Female	3242 (45.6%)	1924 (49.3%)	1318 (41.0%)
Male	3693 (51.9%)	1932 (49.5%)	1761 (54.8%)
Unknown	19 (0.3%)	5 (0.1%)	14 (0.4%)
Race			
American Indian or Alaska Native	22 (0.3%)	13 (0.3%)	9 (0.3%)
Asian	438 (6.2%)	304 (7.8%)	134 (4.2%)
Black or African American	515 (7.2%)	176 (4.5%)	339 (10.6%)
Native Hawaiian or Other Pacific Islander	25 (0.4%)	17 (0.4%)	8 (0.2%)
Other Race or Mixed Race	2048 (28.8%)	987 (25.3%)	1061 (33.0%)
White	3164 (44.5%)	1979 (50.7%)	1185 (36.9%)
Unknown^a^	899 (12.6%)	424 (10.9%)	475 (14.8%)
Ethnicity			
Hispanic	1877 (26.4%)	932 (23.9%)	945 (29.4%)
Non-Hispanic	4237 (59.6%)	2522 (64.7%)	1715 (53.4%)
Unknown/Missing	997 (14.0%)	446 (11.4%)	551 (17.2%)

^a^Note: Unknown race means that a patient cannot or declines to state.

### Prospective use of the VAR model

**Fig. 1:**
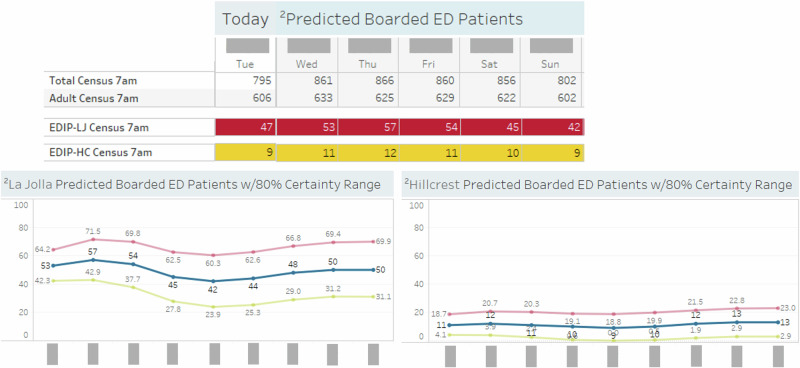
Daily forecasting email for operational decision support. Color-coding of the values is based on operational thresholds set for the La Jolla (LJ) and Hillcrest (HC) emergency departments for different escalations. EDIP refers to emergency department inpatients (i.e., boarding ED patients).

Of the six prospectively evaluated models, only vector autoregression (VAR) was implemented for live operational use. VAR was chosen for its interpretability, stable performance, and capacity to model multivariate dependencies among boarding volume, surgical activity, and hospital census. Each morning at 07:10, after automated data-quality checks, four-day-ahead forecasts (T + 1 to T + 4) were generated and emailed to Mission Control staff and health-system leaders (Fig. 1). Forecasts were reviewed in the daily huddle and considered alongside other indicators to inform same-day actions, including discharge planning, intrasystem transfers, and surgical-case scheduling. This process enabled operational planning up to four days in advance and supported proactive rather than reactive decision-making. Forecasts from the other models were computed concurrently for research comparison but were not disseminated or used in real-time decisions.

### Model performance

**Fig. 2:**
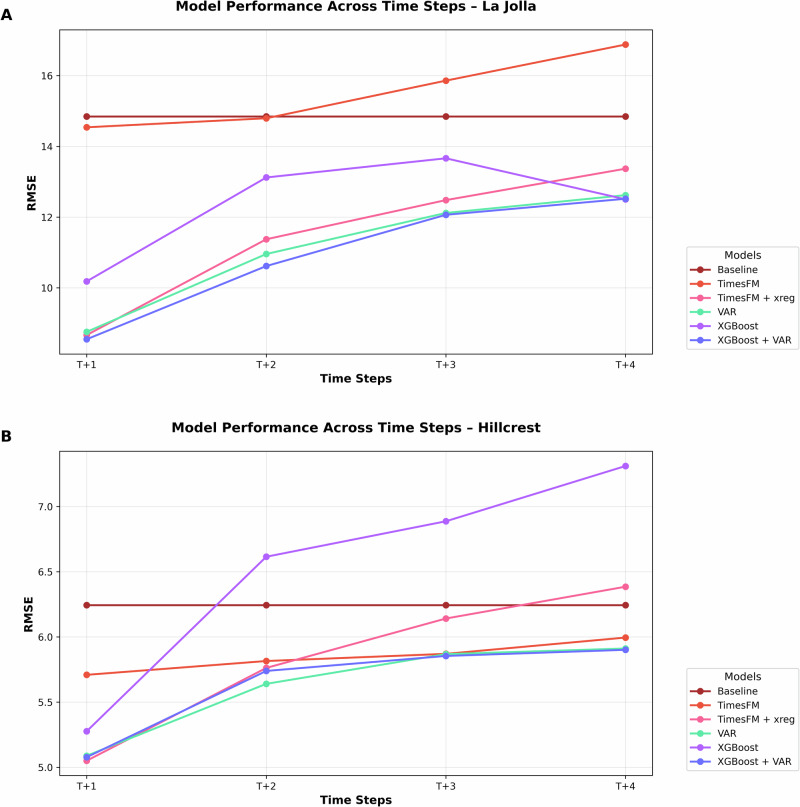
Forecasting model performance across time steps (July–October 2024). Panels **A** and **B** show the root mean squared error (RMSE) for six forecasting approaches across horizons T + 1 to T + 4 at the La Jolla and Hillcrest emergency departments, respectively.

During the four-month validation period, across all six models evaluated, the baseline moving-average model had a root mean squared error (RMSE) of 14.9 for La Jolla and 6.2 for Hillcrest when predicting boarding volumes for horizons T + 1 through T + 4 (Fig. 2A–B**and** Table 2). VAR consistently outperformed the baseline at both sites, demonstrating its ability to capture temporal dependencies and inter-variable relationships. XGBoost performed better than baseline at La Jolla but worse at Hillcrest, indicating site-level variability in nonlinear effects.

**Table 2:** Root Mean Squared Error Comparison by Forecasting Horizon: La Jolla and Hillcrest (July to October, 2024

Model	La Jolla				Hillcrest			
	T + 1	T + 2	T + 3	T + 4	T + 1	T + 2	T + 3	T + 4
Baseline^a^	14.85				6.24			
VAR	8.76	10.96	12.12	12.62	5.09	**5.04**	5.87	5.91
XGBoost	10.18	13.12	13.66	**12.50**	5.28	6.02	6.89	7.31
VAR + XGBoost	**8.55**	**10.62**	**12.07**	12.52	5.08	5.74	**5.85**	**5.90**
TimesFM	14.54	14.79	15.36	16.88	5.71	5.82	5.87	6.00
TimesFM + XReg	8.66	11.37	12.48	13.37	**5.05**	5.76	6.14	6.38

^a^Note: The baseline error was the same for all time horizons because the moving average was calculated based on the preceding two weeks’ values and was thus unchanged for up to T + 6.

The lowest point estimate for error at each time horizon is depicted in bold.

The hybrid VAR+XGBoost combined model performed better than the baseline model at both La Jolla and Hillcrest. VAR+XGBoost had the lowest RMSE for three of four forecast horizons at La Jolla and for two of four horizons at Hillcrest. The VAR+XGBoost model outperformed XGBoost alone at both sites.

TimesFM model performed worse than baseline at La Jolla and better than baseline at Hillcrest. Incorporating covariates in the TimesFM + XReg variant improved accuracy relative to TimesFM, across all forecast horizons at La Jolla but only improved the accuracy at shorter horizons (T + 1 and T + 2) at Hillcrest.

### Uncertainty of VAR predictions

**Fig. 3:**
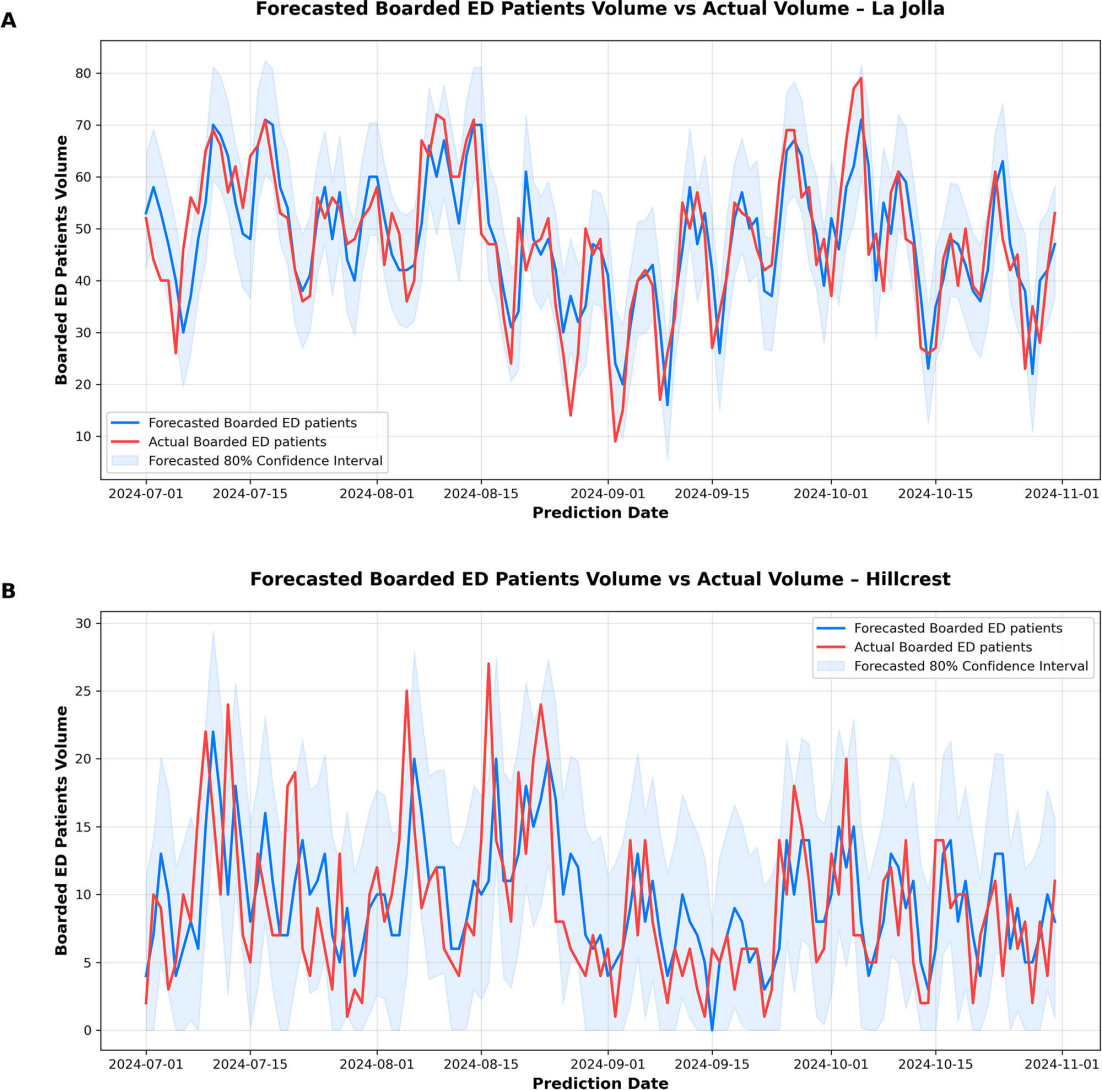
Forecasted versus actual ED boarding volumes using the VAR model. Panels **A** and **B** show predicted boarding volumes (blue line) and observed volumes (red line) for the La Jolla and Hillcrest emergency departments, respectively. The shaded regions represent the 80% confidence intervals.

As described above, the VAR model was in live prospective use as part of clinical workflows during the study period. Figures 3A and 3B depict forecasted ED boarded patient volumes versus actual volumes for La Jolla and Hillcrest, respectively, over the evaluation period, along with 80% confidence intervals. The blue line represents forecasts generated by the VAR model, while the red line indicates actual patient volumes. The shaded regions represent the 80% confidence intervals of the VAR model predictions. Most observed values fell within these intervals, indicating that the model’s uncertainty estimates were well calibrated. Occasional deviations outside the intervals, as shown in Fig. 3, were modest and infrequent, suggesting appropriate coverage of forecast uncertainty during prospective use.

### Monthly variation in performance

As shown in Figs. S1 and S2, RMSE fluctuated substantially across the four-month validation period. Tables S1 and S2 report monthly RMSE and MAE values for each forecast horizon during each month of the validation period, whereas Fig. 2 and Table 2 display RMSE aggregated across the full four-month validation period for each forecast horizon. The lowest baseline RMSE was observed in September at La Jolla and in October at Hillcrest. When compared with each site’s highest monthly RMSE, the lowest monthly baseline RMSE was 22.3% lower at La Jolla and 50.6% lower at Hillcrest (Tables S1 and S2).

## Discussion

Our study demonstrates that VAR-based models, with and without XGBoost, consistently outperform both the previously used moving average model and standalone machine learning approaches in forecasting ED boarding volumes, particularly for short-term time horizons. Over the four-month evaluation period, the VAR models had stable and reliable forecasting accuracy, with significantly lower RMSE and MAE values compared to baseline models. The VAR+XGBoost had the lowest error among all models in our validation set for three out of four time horizons at La Jolla, and two out four time horizons at Hillcrest. This supports the VAR model’s ability to capture temporal dependencies and recurring weekly patterns, enabling adoption to fluctuations in boarding volumes across different months and operational settings. While the TimesFM model underperforming VAR is unsurprising given that TimesFM was not trained on our data (i.e., it is a zero-shot method), its performance below a two-week moving average at La Jolla was unexpected. Adding covariates via TimesFM+XReg improved the TimesFM predictions at La Jolla but not at Hillcrest. XGBoost alone demonstrated modest performance at La Jolla and poor performance (worse than baseline) at Hillcrest. By retraining daily and integrating forecasts into Mission Control huddles, this work illustrates an early step towards a learning health system approach in which data, predictive models, and operational feedback are continuously combined to improve responsiveness.

Although both EDs have high volumes, they differ in several characteristics that may explain why certain approaches worked better at La Jolla versus Hillcrest. In addition to differences in patient populations across the two EDs (Table 1), the average number of boarding patients is several-fold higher at La Jolla with greater variability. The higher variability at La Jolla reflects from differences in referral patterns and surgical volumes. Whereas most patients present to Hillcrest ED on their own, a greater proportion of La Jolla ED patients are referred from clinic, producing weekday-weekend fluctuations. Surgical volumes are also higher at La Jolla and account for a high proportion of inpatients, directly influencing the bed availability for admitted ED patients and affecting their boarding status. These two factors more heavily influence predictions at La Jolla, and thus the models including covariates (VAR, VAR+XGBoost, and TimesFM+XReg models) outperform models without covariates at La Jolla. At Hillcrest, where the covariates are not as important, more parsimonious models (e.g., TimesFM) perform competitively. XGBoost likely performs the worst at Hillcrest because it does not directly model temporal trends and may have overfit to the covariates.

Our study introduces a VAR+XGBoost hybrid framework that systematically integrates econometric multivariate forecasting with machine learning and represents the first clinical application of TimesFM foundation models to ED boarding volume prediction. While the VAR + XGBoost hybrid achieved the lowest overall RMSE, its improvement over VAR alone was modest, underscoring that classical econometric approaches such as VAR remain highly effective for structured, interdependent operational data where linear relationships are prominent. Prior research has focused predominantly on forecasting emergency department arrivals rather than boarding volumes, with most studies using univariate time-series methods such as ARIMA, machine-learning approaches, or feature-engineering frameworks for arrival prediction^21–24^. In contrast, few studies have leveraged vector autoregression and foundation-model architectures for prospective forecasting of boarding volumes. Our systematic comparison of standalone econometric, machine-learning, foundation model, and hybrid VAR + XGBoost approaches for ED boarding volume prediction demonstrated substantially higher forecasting accuracy—achieving a 41% reduction in RMSE compared with baseline, exceeding the typical 10–30% gains reported for arrival forecasting—thereby underscoring the clinical utility of integrating econometric and machine-learning methods for operational decision support^23,24^.

Our study has limitations. Because the VAR model was in live use during the study period, it is possible that the predictions could have influenced health system ED boarding behavior, introducing a feedback loop. In practice, such a feedback loop could have led the Mission Control team to take actions affecting the number of boarding ED patients. However, Fig. 3A, B show that the VAR model remained well-calibrated, suggesting that any feedback effects were limited. Another limitation is that we did not directly evaluate the influence of the implemented VAR model on reducing boarding volume. Because Mission Control was launched in April of 2024, the workflows to act on model predictions were not yet finalized during the study period. Operational workflows directly making use of model predictions have recently been established, and an evaluation of operational effectiveness is underway. Beyond accuracy metrics, future work could also evaluate the operational value of forecasting by integrating predictive models into simulation-based or stochastic optimization frameworks, as demonstrated by Mišić et al.^25^. Such approaches could quantify the potential impact of short-term forecasts on capacity planning, staffing, and elective-surgery scheduling decisions.

Despite with these limitations, our findings highlight the promise of VAR-based forecasting methods for managing ED boarding volumes, with further nonlinear trends captured through the VAR+XGBoost hybrid approach. This study demonstrates the feasibility of integrating system-level forecasts into operational workflows and retraining models daily to reflect changing hospital dynamics. Future work should determine whether such forecasting approaches can directly reduce ED boarding times and improve patient flow outcomes. Daily health system-level communications and interventions coordinated by a centralized Mission Control team provide the basis for an at-scale implementation that will serve as the basis for future impact evaluation.

## Methods

### Study setting

This prospective study analyzed data collected from two EDs within UC San Diego Health: those on the La Jolla and Hillcrest campuses. The La Jolla ED serves the Jacobs Medical Center and Sulpizio Cardiovascular Center at UC San Diego Health and is home to California’s first accredited geriatric emergency department. Its 24/7 emergency department manages approximately 40,000 visits annually, with volumes varying by season and time. The Hillcrest ED at UC San Diego Health is a Level 1 Trauma Care Center and provides critical acute care to a diverse population, including underserved communities, with its busy 24/7 emergency department handling approximately 44,000 visits annually.

The study protocol was reviewed and approved by the UCSDH ACQUIRE committee, which classified the study as not involving human subjects under regulatory guidelines. Given the aggregated nature of the data and absence of patient-specific identifiers, informed consent requirements were waived.

### Data extraction and allocation

Data features were selected through a rigorous process involving feature importance analysis, consultations with experts, key stakeholders, and insights gathered from daily Mission Control huddles, ensuring alignment with operational priorities and clinical relevance.

Data extraction was conducted using automated daily queries via UC San Diego Health’s Tableau and Epic Clarity platforms to ensure consistency and completeness. The following variables were extracted: daily ED boarding volume at midnight and 7 am; daily planned surgical volume; total hospital volume; and temporal indicator variables (e.g., weekday vs. weekend).

Daily data from October 1, 2022, through October 31, 2024, were extracted for model development and validation. Data from October 1, 2022, through June 30, 2024, were used only for model development, whereas data from July 1, 2024, through October 31, 2024, were used for both model development and validation. As described further below, models were retrained each day using data up through time T, with T + 1 through T + 4 used for validation.

### Data preprocessing

Missing values were rare ( < 1%) during initial quality assessments. Accuracy of the data elements had previously been verified via their use in clinical operational workflows. Accuracy of timestamps was verified through both manual and automated review. The dataset was structured in a matrix format suitable for time series forecasting, with each row representing a single day, and columns representing each variable. Temporal indicators, such as weekday and weekend classifications, were retained as binary variables.

### Model development

To forecast ED boarding volumes, we prospectively evaluated six models spanning three methodological families: an econometric model (vector autoregression [VAR]), a machine-learning model (extreme gradient boosting [XGBoost]), and a time-series foundation model (Google TimesFM), including two extensions (VAR + XGBoost and TimesFM + XReg) and the two-week moving-average operational baseline. All models followed a common framework: each morning, models were re-estimated using a rolling window of all data through day *T* and generated four-day-ahead forecasts (T + 1 to T + 4). The retraining schedule, forecast horizons, and evaluation metrics (RMSE and MAE) were kept identical across methods to enable a fair, head-to-head comparison.

#### Baseline moving average

A two-week moving average was used as the baseline model because it was the default option in use by the Mission Control team within the Epic electronic health record.

#### Vector autoregression

The vector autoregression (VAR) model is an econometric model well-suited for capturing multivariate temporal dynamics and analyzing interdependencies among multiple endogenous variables^26^. The model is particularly suitable for addressing the dynamic nature of ED patient flows^27,28^. Unlike traditional methods, this VAR-based forecasting model integrates real-time and historical data to address the specific demands of ED boarding, allowing hospitals to implement timely interventions. It extends univariate autoregression to multivariate time series, allowing each variable to be a linear function of past values of itself and other variables. The model is formally expressed as:

1 $${Y}_{t}=c+{A}_{1}{Y}_{t-1}+{A}_{2}{Y}_{t-2}+\ldots +{A}_{p}{Y}_{t-p}+{\epsilon }_{t}$$

where:

- ${Y}_{t}$ represents the vector of variables at time t
- $c$ is a vector of constants
- ${A}_{i}$ are coefficient matrices
- $p$ is the lag order
- ${\epsilon }_{t}$ is the error term

We empirically determined the lag order $(p)$ by testing values from 1 to 10 days. We found that a 4-day lag demonstrated optimal performance in minimizing the root mean squared error (RMSE) and effectively captured temporal dependencies and recurring weekly patterns in ED boarding volumes. This selection was also supported by the Akaike information criterion (AIC) and Bayesian information criterion (BIC).

We employed a rolling window approach using the complete historical dataset. Each day at 7:10 AM, after integrating the latest ED boarding volumes, the model was retrained to generate forecasts for the next four days ($T+1$ to $T+4$). The coefficient matrices were re-estimated each morning using all data through day *T*. Although less frequent retraining was considered, empirical tuning indicated that daily updates better captured short-term dependencies. This approach allowed the model to incorporate recent trends not present in earlier training data. The training process focused on minimizing RMSE to optimize parameters and coefficients.

#### Extreme gradient boosting

The XGBoost model is an ensemble learning method recognized for modeling nonlinear relationships and complex patterns, with successful application to time-series data^29^. It uses gradient boosting algorithms to enhance predictive performance by combining multiple weak learners into a strong predictor. We trained XGBoost using the same input features as the VAR model to capture potential nonlinearities in the data, incorporating 2 weeks of lagged variables to provide sufficient historical context for prediction. The model was implemented with 100 boosting rounds, a learning rate of 0.1, histogram-based tree construction, and early stopping after 5 rounds without improvement (minimum delta = 0.001) to prevent overfitting.

#### Time series foundation model

TimesFM is a foundation model developed by Google Research for univariate time-series forecasting. It relies on a patched decoder attention architecture that is pretrained on diverse datasets^30^. While TimesFM does not directly support the use of covariates for forecasting, the TimesFM+XReg extension to this method includes covariates as batched in-context exogenous regressors (XReg). The TimesFM+XReg method fits linear models on each covariate, and the final forecast is generated by combining the TimesFM forecast with the predictions from the linear models. However, due to the model architecture, covariate values must be assumed to be known in advance; consequently, additional methods are required to forecast the covariates before incorporating them into the forecasting process.

#### Novel approach—hybrid VAR + XGBoost model

For a rigorous assessment, we explored combining the VAR model with XGBoost. The rationale behind using this model combination is that XGBoost is not structurally designed to directly handle time-series data. In contrast, the VAR model, with its multivariate nature, possesses the ability to forecast covariates and utilize them for target forecasting. By integrating VAR’s strengths in multivariate forecasting with the capability to model non-linear data of XGBoost, we aimed to improve the overall accuracy of ED boarding volume predictions.

In the VAR+XGBoost implementation, the VAR model first generated forecasts for all covariates at each time step, which were then used as input features for XGBoost to predict ED boarding volumes. This sequential approach allows the hybrid model to maintain temporal dependencies in the covariates while leveraging XGBoost’s advanced pattern recognition capabilities for the final prediction.

### Model validation

We evaluated all six forecasting models by assessing predictive accuracy over a four-day forecasting horizon using RMSE. Evaluations at each forecast interval ($T+1$ to $T+4$) provided quantitative assessments of forecasting accuracy, with lower values indicating superior performance. To ensure real-world applicability, we implemented a weekly manual review process. Model performance is monitored, and necessary adjustments are documented and implemented, ensuring alignment with current ED boarding volume trends and operational demands. By exploring these models and their combinations, we aimed to enhance predictive performance and leverage the complementary strengths of different methods within the context of ED data. This systematic comparison enabled us to evaluate the potential improvements introduced by integrating the VAR model with various established forecasting methods, assessing their efficacy in predicting ED boarding volumes.

### Software

We used Python 3.11 for analysis, with statsmodels, xgboost, and timesfm used for implementations of VAR, XGBoost, TimesFM, and TimesFM+XReg.

## Supplementary information

Supplementary Information

## Data Availability

The data used in this study were obtained from UC San Diego Health and analyzed within secure institutional environments. These data are not publicly available because they contain protected health information, and restrictions apply to their use.

## Code availability

The code used in this study is available from the corresponding author upon request.
