# Supplementary material for: Prospective comparison of econometric, machine learning, and foundation models for forecasting emergency department boarding patients
Source: Npj Health Syst. 2025 Dec 16;2:49. doi: 10.1038/s44401-025-00054-z (PMC13354169; doi:10.1038/s44401-025-00054-z)

**Supplementary Materials**

**Tables**

Table S1. Performance Comparison of Various Models Across Months and Time Steps – La Jolla

Table S2. Performance Comparison of Various Models Across Months and Time Steps – Hillcrest

**Figures**

Figure S1. RMSE Comparison Across Months for Time Steps – La Jolla


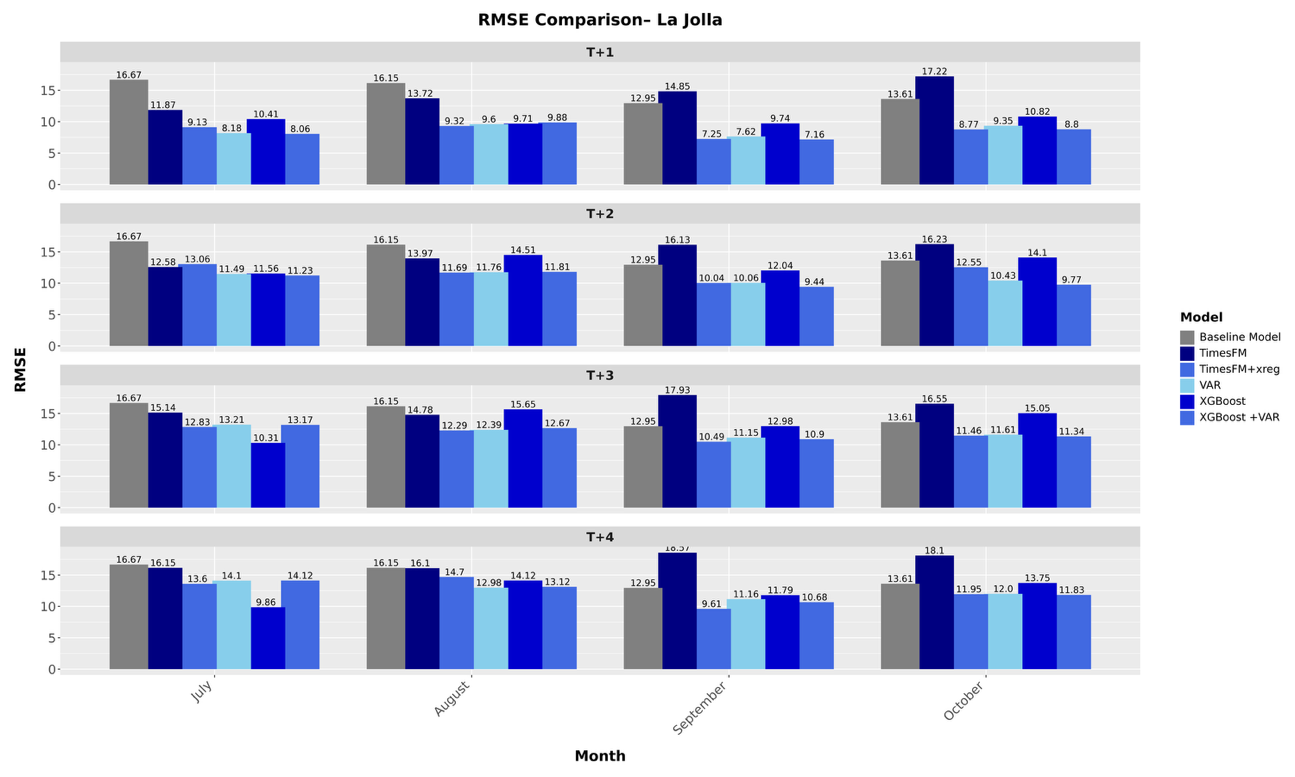


Figure S2. RMSE Comparison Across Months for Time Steps – Hillcrest


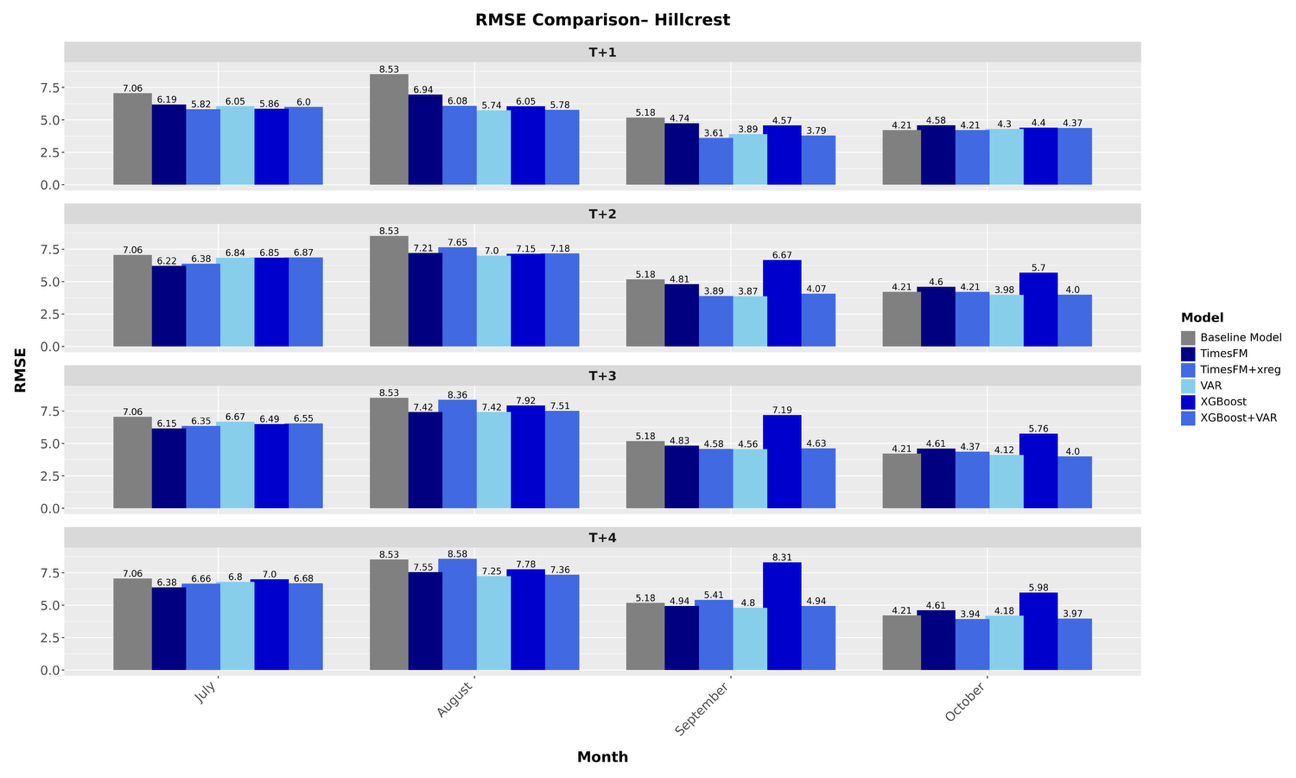

Supplement: Supplementary file 1 — Supplementary Information [file 44401_2025_54_MOESM1_ESM.docx]
